# Malaysian Public Interest in Common Medical Problems: A 10-Year Google Trends Analysis

**DOI:** 10.7759/cureus.21257

**Published:** 2022-01-14

**Authors:** Ren Yi Kow, Norfazilah Mohamad Rafiai, Akmal Azim Ahmad Alwi, Chooi Leng Low, Nur Raziana Rozi, Khairul Nizam Siron, Ahmad Hafiz Zulkifly, Zamzuri Zakaria@Mohamad, Mohamed Saufi Awang

**Affiliations:** 1 Department of Orthopaedics, Traumatology & Rehabilitation, International Islamic University Malaysia, Kuantan, MYS; 2 Department of Education and Research, Sultan Ahmad Shah Medical Centre@IIUM (International Islamic University Malaysia), Kuantan, MYS; 3 Department of Plastic and Reconstructive Surgery, International Islamic University Malaysia, Kuantan, MYS; 4 Department of Radiology, Sultan Ahmad Shah Medical Centre@IIUM (International Islamic University Malaysia), Kuantan, MYS; 5 Department of Internal Medicine, International Islamic University Malaysia, Kuantan, MYS; 6 Department of Neurosurgery, Sultan Ahmad Shah Medical Centre@IIUM (International Islamic University Malaysia), Kuantan, MYS

**Keywords:** language of medicine, public healthcare, malaysia, google trends healthcare, google trend

## Abstract

Background

An analysis of internet search has been performed to evaluate the public interest in health problems. Google Trends (GT) serves as a free platform to analyse the search traffic for specific terms in the Google search engine. This observational study aims to investigate the trend of Malaysian population in using the Google search engine on common medical problems and explore the geographical influence on the language used.

Material and method

Fifteen pairs of keywords, in Malay and English language, were chosen after going through forward and backward translation and vetting by a panel of experts. GT data for the selected keywords from 1st of January 2011 to 31st of December 2020 was extracted. Trend analysis was performed using paired t-test between the first half of the decade and the second half of the decade. The different languages used were analysed based on geographical variation using paired t-test.

Results

The public interest on those keywords was markedly increased in the second half of the decade with 29 out of 30 keywords showing statistically significant difference. Majority of the states preferred to use Malay keywords, especially those residing at the East Coast of Peninsular Malaysia.

Conclusion

This observational study illustrates the ability of GT to track healthcare interest among Malaysian population. GT provides a good platform to analyse specific healthcare interest in Malaysian population, but investigators have to bear in mind the geographical influence on the language used.

## Introduction

As the internet has become more accessible, many people are obtaining information from internet sources [1,2]. As the most popular search engine currently, the Google search has become an important source of information for many people [3-5]. Lately, researchers have been capitalizing on this trend to obtain data on topics of interest, ranging from deep learning to currency exchange rate [1,2]. Similarly, the Google search has become a source of health information to lay persons and healthcare personnel alike [6-9]. By analysing the trend of Google search, the public interest in various medical problems can be assessed, giving valuable information for proper planning and funding allocation in healthcare [10-13]. Google Trends (GT) is a tool that allows users to freely access Google search data. It provides an in-depth analysis of billions of daily Google search results and provides information on geographical and temporal patterns in search volumes for user-specific terms.

Recently, the analysis of Google Trends has been utilised with success in Malaysia in multiple medical fields, ranging from analysis of COVID-19 to breast cancer [13-15]. By comparing the search volume index (SVI), a geographical difference can be determined, making funding allocation much easier [10-15]. Furthermore, the language used for Google search can be assessed using the GT. This is especially important in a multi-racial, multi-lingual country like Malaysia. Despite GT is being utilised throughout the medical field, thus far there is no GT analysis that investigates the language difference in Google search of common medical problems in Malaysia. We aim to conduct a cross-section observational study to determine the public interest in common medical problems in Malaysia and the language difference in Google search of these medical problems over a 10-year period.

## Materials and methods

There were two parts in this study, namely i) determining the content-validated English and Malay search keywords and, ii) applying Google Trends to assess the search volume index (SVI) of a specific term per time point in relation to the total number of searches in the Google search engine. Ethical clearance and consent were not required as this study did not involve human participants and the data was freely available online.

**Figure 1:**
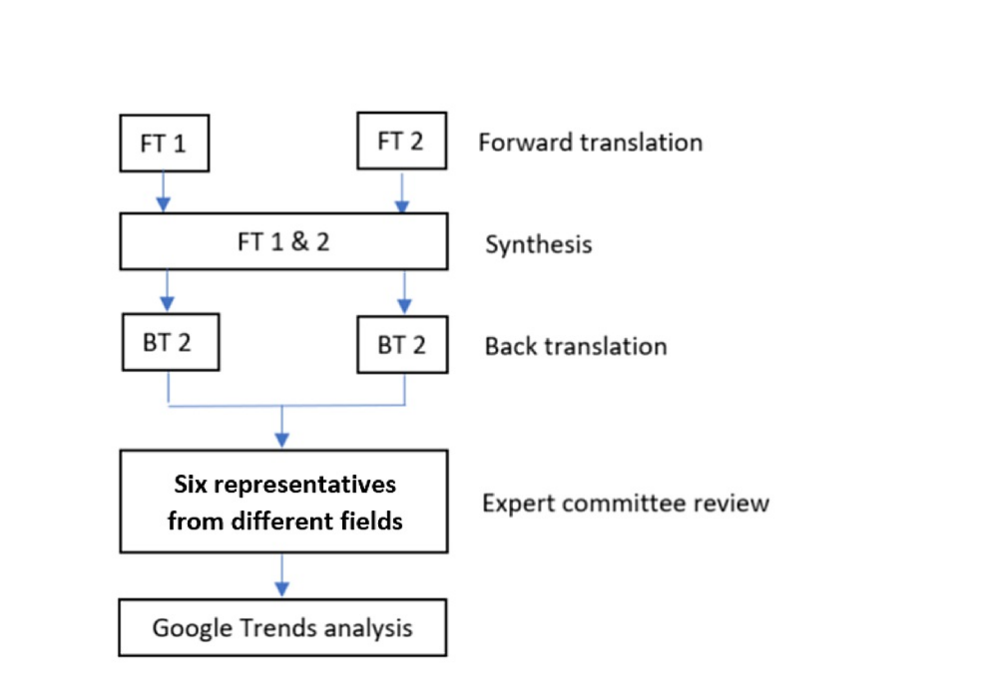
The flow of keywords selection was summarized. All keywords went through forward and backward translations before being reviewed by the expert committee. Only keywords that had achieved an item-level content validity index (I-CVI) of 1.00 were included in the subsequent analysis using Google Trends.

In the first part, an expert team was formed by six representatives from different fields including the deputy rector, hospital director, orthopaedic, radiology, medical and surgical departments to validate the keywords. Paired keywords in both English and Malay languages were proposed to the expert team after going through forward and backward translations. Only keywords that had achieved an item-level content validity index (I-CVI) of 1.00 were included in the subsequent analysis using GT [16]. A total of 15 pairs of keywords attained I-CVI of 1.00 from 1st of January 2011 to 31st of December 2020 and they were analysed using GT. The year 2011 was chosen as this was the time when more than 80% of users started using Google search rather than other search engines such as Yahoo and Bing [17]. The methodology is summarized in Figure 1.

**Figure 2:**
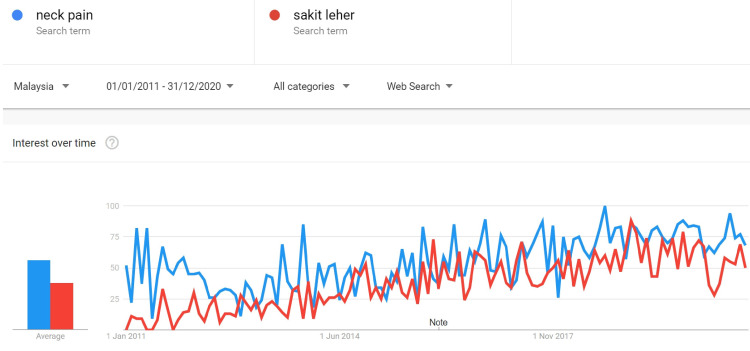
The comparison between keywords “neck pain” and “sakit leher” was shown. Both keywords showed an increasing trend on Google search.

**Figure 3:**
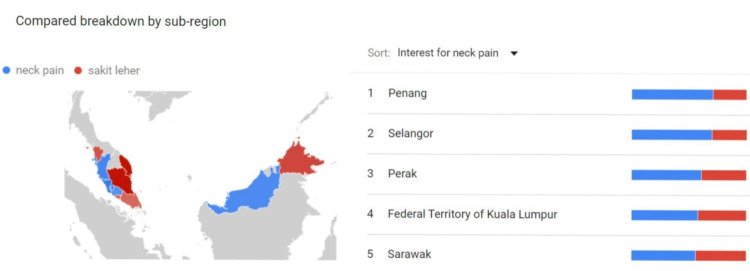
Geographical mapping of the language used for Google search. For instance, Penang, Selangor, Perak, Kuala Lumpur and Sarawak showed a predominant English usage for this paired keyword.

In the second part, we applied GT’s customizable geographic and temporal filters to include results for searches within Malaysia from 1st of January 2011 to 31st of December 2020. After the selected search terms were entered into the GT system, GT would summarize outputs that described the frequency of searches for a given search term relative to the maximum popularity within the selected time [10]. This was called the search volume index (SVI) and it reflected the popularity of a specific search term in relation to the total volume of search queries for that specific geographical location and time [12]. The monthly SVI score ranged from 0 to 100, whereby a score of 0 reflected no search for that specific term in that month and a score of 100 represented the highest monthly search for that specific term during the study period [10,12]. In order to evaluate the trend of Google searches in Malaysia, we had extracted the monthly SVI score for each of the selected keyword. The extracted scores were tabulated and inserted into SPSS version 21.0 (IBM Corp., Armonk, NY) and the mean score for the first five years (from January 2011 to December 2015) was compared with the mean score for the subsequent five years (from January 2016 to December 2020) by using paired t-test. Next, we investigated the Google search difference between English and Malay languages for all the selected keywords. Paired keywords were entered into the GT as shown in Figure 2. The geographical influence on the language used was assessed by tabulating geographical scores of all 15 paired keywords (Figure 3) and an analysis using paired t-test was performed.

## Results

Trend in Google search for common medical problems

Table 1 summarises the mean SVI comparison between the first five years (from January 2011 to December 2015) and the second five years (from January 2016 to December 2020) for all 15 pairs of keywords. All keywords showed an upward trend of SVI in the second half of the decade compared to the first half of the decade. The increase of SVI ranged from 3.59 for “Hypertension” to 40.81 for “Sakit kepala”. The SVI increment within this period was statistically significant for all bar one keyword “Hypertension”.

**Table 1:** The mean SVI comparison between the first 5 years (from January 2011 to December 2015) and the second 5 years (from January 2016 to December 2020) for all 15 pairs of keywords. SVI: search volume index *Student t-test

Keywords	Mean (1st 60 months)	Mean (2nd 60 months)	Difference	P-value*
Headache	51.87	74.73	+22.86	<0.001
Sakit kepala	38.77	79.58	+40.81	<0.001
Cough	41.80	57.05	+15.25	<0.001
Batuk	36.97	67.52	+30.55	<0.001
Dizzy	51.72	69.18	+17.46	<0.001
Pening	43.48	83.78	+40.30	<0.001
Red eye	44.18	54.22	+10.03	<0.001
Mata merah	32.77	61.12	+28.35	<0.001
Chest pain	45.87	57.37	+11.50	<0.001
Sakit dada	27.55	56.33	+28.78	<0.001
Neck pain	35.23	58.87	+23.64	<0.001
Sakit leher	20.97	54.73	+33.76	<0.001
Back pain	48.48	71.70	+23.22	<0.001
Sakit belakang	39.92	78.17	+38.25	<0.001
Knee pain	41.75	61.45	+19.70	<0.001
Sakit lutut	39.67	71.85	+32.18	<0.001
Diabetes	60.93	71.63	+10.70	<0.001
Kencing manis	58.00	80.38	+22.38	<0.001
Hypertension	67.88	71.47	+3.59	0.095
Darah tinggi	47.40	76.20	+28.80	<0.001
Cancer	52.30	61.73	+9.43	<0.001
Kanser	24.78	37.67	+12.89	<0.001
Fever	62.73	69.50	+6.77	<0.001
Demam	42.08	65.47	+23.38	<0.001
Vomit	49.57	62.22	+12.65	<0.001
Muntah	34.92	68.37	+33.45	<0.001
Diarrhea	54.32	72.25	+17.93	<0.001
Cirit	37.20	70.93	+33.73	<0.001
Abdominal pain	51.83	67.28	+15.45	<0.001
Sakit perut	44.02	84.55	+40.53	<0.001

Geographical influence on the language usage

Table 2 summarises the comparison of mean SVI between English and Malay languages in different states of Malaysia. Out of the different states studied, the majority of the states (13 out of 16 states) showed a predominant Malay language usage. The difference of mean SVI ranged from 4.00 in Kuala Lumpur to 38.40 in Terengganu. Only three states demonstrated a higher rate of English usage, namely Penang, Selangor, and Sarawak. The mean SVI differences were 17.46, 13.33 and 0.94 for the three states, respectively. In Malay language predilection areas, the differences were statistically significant in Kedah, Kelantan, Pahang, Perlis and Terengganu. Only Penang state exhibited a statistically significant difference in the English inclination area.

**Table 2:** The comparison of mean search volume index (SVI) between English and Malay languages in different states of Malaysia. *Student t-test Language predominance is highlighted in bracket. For example, 4.00 (M) in Kuala Lumpur represents a Malay language predominance in that state. Similarly, 13.33 (E) in Selangor represents an English language predominance in Selangor.

States	Mean (English)	Mean (Malay)	Difference	P-value*
Kuala Lumpur	48.00	52.00	4.00 (M)	0.654
Johor	43.87	56.13	12.26 (M)	0.222
Kedah	33.53	66.47	32.93 (M)	0.007
Kelantan	29.47	63.87	34.40 (M)	0.009
Labuan	27.80	32.20	4.40 (M)	0.786
Malacca	39.13	60.87	21.73 (M)	0.062
Negeri Sembilan	42.60	57.40	14.80 (M)	0.170
Pahang	32.67	67.33	34.67 (M)	0.008
Penang	58.73	41.27	17.46 (E)	0.038
Perak	44.27	55.73	11.47 (M)	0.200
Perlis	24.67	55.33	30.66 (M)	0.036
Putrajaya	30.60	49.40	18.80 (M)	0.210
Sabah	42.40	57.60	15.20 (M)	0.109
Sarawak	50.47	49.53	0.94 (E)	0.912
Selangor	56.67	43.33	13.33 (E)	0.113
Terengganu	24.13	62.53	38.40 (M)	0.004

## Discussion

As the internet has become increasingly accessible in Malaysia, people turn to the web to seek information regarding their medical problems. In this study, we have captured the increasing popularity of Google search as a tool to obtain information on common medical problems in Malaysia. The heavy GT traffic in the second half of the decade reflects the willingness of the Malaysian population to use Google search. The surge of GT traffic is demonstrated in both English and Malay keywords across Malaysia. Likewise, Google searches have increased in all keywords across different disciplines in medicine, be it medical-based or surgical-based. This upward trend is consistent with other studies in the literature [9-15]. For example, Lim et al. reportedly used GT to track Malaysian public information-seeking behavior while Mohamad and Kok used GT to track public interest in breast cancer screening in Malaysia [14,15].

In this study, we have explored the geographical influence on the language usage in Google search. With Malay ethnicity as the major population and Malay language being the national language in Malaysia, it is unsurprising that the majority of the states use Malay keywords more frequently than English keywords. The difference is more pronounced in the East Coast of Malaysia with Terengganu, Kelantan and Pahang occupying the top three of the chart in terms of Malay language usage. On the other hand, only Penang state exhibits statistically significant English usage compared to the Malay language. The presence of various missionary schools, international schools, colleges, and universities may have contributed to the language used in this state.

Characterization of the link between Google searches and healthcare is important in identifying the demand of the target population. By harvesting the GT tool to track the healthcare interest among the Malaysian population, we can anticipate and mobilize crucial resources to on-demand disciplines. Financial allocation and training of new staff can be planned if the healthcare demand can be predicted. In the same vein, geographical variation has to be taken into account as human resources need to be trained to be able to effectively convey health information to the population in their preferred languages.

Limitation

There are several limitations in this study. First of all, the 15 pairs of keywords are selected by the expert panel to capture interest across different disciplines in medicine. Nevertheless, this list is not exhaustive and may not be representative for niche area such as rare diseases. Similarly, the language predominance may be affected by the use of different keywords. Nonetheless, this is the first study that shows a geographical influence on the language used in Google search. Besides that, there are different keywords which may have multiple synonyms and translations. For example, SVI for keywords such as “cancer”, “malignancy” and “tumour” that describe a similar disease may be different. In the same vein, keywords such as “kanser”, “barah”, and “ketumbuhan” may be similar in Malay language and they may affect the keyword SVI. However, we try to limit the discrepancy by performing forward and backward translations for the keywords prior to vetting by the expert panel. Lastly, the upward trend of GT may be partially explained by the increasing number of Malaysians who have access to the internet throughout the study period.

## Conclusions

In conclusion, our study demonstrates an upward trend in GT search volumes of common medical problems in Malaysia. Besides that, geographical variation influences the language used in Google search and the Malay language is the preferred language in the majority of the states in Malaysia. The link between Google search and the interest of the public can be delineated by analysing the GT. By analysing the vast data available in GT, the health authority can plan and allocate their resources to on-demand areas.
